# Implementation and facilitation of post-resuscitation debriefing: a comparative crossover study of two post-resuscitation debriefing frameworks

**DOI:** 10.1186/s12873-022-00707-4

**Published:** 2022-09-02

**Authors:** April J. Kam, Clarelle L. Gonsalves, Samantha V. Nordlund, Stephen J. Hale, Jennifer Twiss, Cynthia Cupido, Mandeep Brar, Melissa J. Parker

**Affiliations:** 1 Department of Pediatrics, Division of Pediatric Emergency, McMaster University, McMaster Children’s Hospital, 1200 Main Street West, Hamilton, ON L8N 3Z5 Canada; 2 Department of Pediatrics, Hospital for Sick Children, 555 University Avenue, Toronto, ON M5G 1X8 Canada; 3 Department of Pediatrics, Division of Pediatric Endocrinology, McMaster University, 1280 Main Street West, Hamilton, ON L8S 4L8 Canada; 4 Department of Emergency Medicine, University of Manitoba, Winnipeg, MB R3E 0W2 Canada; 5 Department of Pediatrics, Division of Neonatology, McMaster University, Hamilton, L8N 3Z5 Canada; 6 Department of Pediatrics, Division of Pediatric Critical Care, McMaster University, McMaster Children’s Hospital, 1200 Main Street West, Hamilton, ON L8N 3Z5 Canada; 7 Emergency Department, McMaster Children’s Hospital, 1200 Main Street West, Hamilton, ON L8N 3Z5 Canada

**Keywords:** Debriefing, Pediatric, Emergency medicine, Resuscitation

## Abstract

**Background:**

Post-resuscitation debriefing (PRD) is the process of facilitated, reflective discussion, enabling team-based interpersonal feedback and identification of systems-level barriers to patient care. The importance and benefits of PRD are well recognized; however, numerous barriers exist, preventing its practical implementation. Use of a debriefing tool can aid with facilitating debriefing, creating realistic objectives, and providing feedback.

**Objectives:**

To assess utility of two PRD tools, Debriefing In Situ Conversation after Emergent Resuscitation Now (DISCERN) and Post-Code Pause (PCP), through user preference. Secondary aims included evaluating differences in quality, subject matter, and types of feedback between tools and implications on quality improvement and patient safety.

**Methods:**

Prospective, crossover study over a 12-month period from February 2019 to January 2020. Two PDR tools were implemented in 8 week-long blocks in acute care settings at a tertiary care children’s hospital. Debriefings were triggered for any intubation, resuscitation, serious/unanticipated patient outcome, or by request for distressing situations. Post-debriefing, team members completed survey evaluations of the PDR tool used. Descriptive statistics were used to analyze survey responses. A thematic analysis was conducted to identify themes that emerged from qualitative responses.

**Results:**

A total of 114 debriefings took place, representing 655 total survey responses, 327 (49.9%) using PCP and 328 (50.1%) using DISCERN. 65.2% of participants found that PCP provided emotional support while only 50% of respondents reported emotional support from DISCERN. PCP was found to more strongly support clinical education (61.2% vs 56.7%). There were no significant differences in ease of use, support of the debrief process, number of newly identified improvement opportunities, or comfort in making comments or raising questions during debriefs between tools. Thematic analysis revealed six key themes: communication, quality of care, team function & dynamics, resource allocation, preparation and response, and support.

**Conclusion:**

Both tools provide teams with an opportunity to reflect on critical events. PCP provided a more organized approach to debriefing, guided the conversation to key areas, and discussed team member wellbeing. When implementing a PRD tool, environmental constraints, desired level of emotional support, and the extent to which open ended data is deemed valuable should be considered.

## Introduction

Post-resuscitation debriefing (PRD) is the process of facilitated reflective discussion in order to allow for both interpersonal feedback within a team and identify systems-level barriers to quality patient care [1]. In the healthcare setting, PRD is usually employed following scenarios which involve team-based work under high stakes and often fast paced environments, such as following a resuscitation or unexpected patient outcome. It allows for the team to come together and reflect upon what was done well and what can be improved upon, thus serving as an important forum for assessment of factors that contribute towards quality improvement and patient safety in real time, with the potential to recognize barriers to care, gaps in training, and propose solutions that can contribute to better patient care and safer work environments. The practice of post-resuscitation debriefing also carriers the additional benefits of providing a forum of peer-based support for healthcare workers following scenarios that are often distressing, and thus acting as a form of wellness support for acute care workers., as well as playing an important role in medical education as it provides a forum for teaching salient points relevant to patient care following high acuity scenarios. PRD is recommended by American, Canadian, and European resuscitation guidelines and has been shown to be beneficial for improving patient outcomes, improving team function, and improving health care provider stress levels and feelings of competency [2–9].

Although the importance and benefits of post-resuscitation debriefing are well recognized, and the salient components of the practice of debriefing are well-described in the literature, a national needs assessment of Canadian Pediatric Emergency Departments found that despite recognizing the need for and importance of post-resuscitation debriefing, the practice of debriefing itself occurs less than 25% of the time,and for the majority of practitioners, there is no institutional expectation that debriefing should occur. Most healthcare providers believed that some sort of formal training was needed to facilitate a debriefing and barriers to debriefings were found to be Emergency Department (ED) workload and time shortages. This study also found that the use of a debriefing tool could aid with facilitating the process, creating realistic objectives, and providing feedback [2].

Despite the practice of PRD being widely recommended, an important challenge presented by the existing PRD literature is the variety of environments in which it has described and studied, ranging from simulation [1], to clinical environments [2–4], to guidelines [5], as well as within the realm of medical education and assessment [6–10]. Although this highlights the multimodal use of PRD in the practice of medicine, there are inherent differences in each of these environments. These differences include outcomes of interest, which may include educational learning outcomes in simulation settings, while team functioning and patient outcomes are a greater focus in clinical settings. Additionally, time restraints are a more important factor in clinical settings. Other factors to consider are that the diversity, familiarity, and number of multidisciplinary team members, all of which can vary significantly from simulation environments to acute care settings. As a result, it is important to clarify the objectives for debriefing as this has the potential to help in identifying both the method(s) and outcomes measures of importance when selecting debriefing tools [11]. With regards to the process of debriefing, Rudolph et al. described an evidence and theory based four step model of debriefing which includes 1) noting salient performance gaps, 2) providing feedback describing the gap, 3) investigating the basis for the gap based on current contributing, and 4) discussion and instruction on how to close the gap [10]. To aid in the process of facilitating and implementing appropriate PRD strategies, Coggin et al. have identified and outlined twelve tips, which highlight the importance of clarifying goals of PRD, ensuring team members recognize its importance, as well as environmental, psychological, and cultural factors [12].

Although various PRD tools exist, a lack of standardized data reporting has made comparison between methods impossible and there is scant research directly comparing debriefing methods. This presents a significant barrier to the widespread adoption of PRD; even if a group is interested and motivated to adopt a formalized PRD framework, the lack of comparative study between methods makes it impossible to make an informed, evidence-based decision on which framework to implement [13, 14]. An additional challenge of the existing PRD literature is the variety of environments in which it has been created, described, and studied, ranging from simulation, to clinical environments, to guidelines, as well as within the realm of medical education and assessment. This highlights the multimodal use of PRD in the practice of medicine but presented challenges when selecting frameworks to use for the purposes of this study. Additionally, the literature on PRD is often split between use in either the ED, part of code teams, or in other acute care environments, or applied broadly across an entire hospital. This raises the question of whether different PRD tools or styles may be more effective in one context compared to others. Previous work by Hale et al. in 2020 sought out to identify debriefing frameworks that are currently used in the Emergency Department through a systematic review, which identified six key frameworks: CCHS, DISCERN, INFO, PediRes-Q, and PCP. PRD is often tasked with the dual role of providing both focused, performance-oriented feedback as well as emotional support for team members. Therefore, it is important that a PRD tool have the optimal balance between a number of competing variables which allow for the tool to be comprehensive and allow for thorough debriefing while also being easy to learn and time efficient in use in order to facilitate widespread adoption. Of the established PRD tools available, two were of particular interest to our team. Debriefing In Situ Conversation after Emergent Resuscitation Now (DISCERN) and Post-Code Pause (PCP) were chosen for the purposes of the present study as these tools represent two different approaches to debriefing [13].

The first being DISCERN, an established, plus-delta based debriefing method. The second PRD tool we assessed was the PCP, which while still rooted in plus-delta principles has a greater focus on health care provider wellbeing and emotional support [15, 16]. DISCERN is a more involved debriefing process that targets QI-type performance improvement, while PCP is a simpler process targeted more at the emotional well-being of care team members [13]. As a result, this study compares the use of two existing but not yet validated debriefing frameworks in multiple acute care settings: the Neonatal Intensive Care Unit (NICU), Pediatric Intensive Care Unit (PICU), Code Blue team, and the Emergency Department (ED) in an academic tertiary care pediatric hospital.

The primary objective of this study was to assess user preference with regards to utility of two PRD tools, DISCERN and PCP, based on personal, situational, environmental, and team-based factors, with the ultimate goal of this initiative being to institute a children’s hospital-wide debriefing tool for PRD. Secondary aims included determining whether there were differences in the quality, subject matter, and types of feedback garnered from these different tools and potential implications on quality improvement and patient safety.

## Materials and methods

### Study design and timeline

**Fig. 1:**
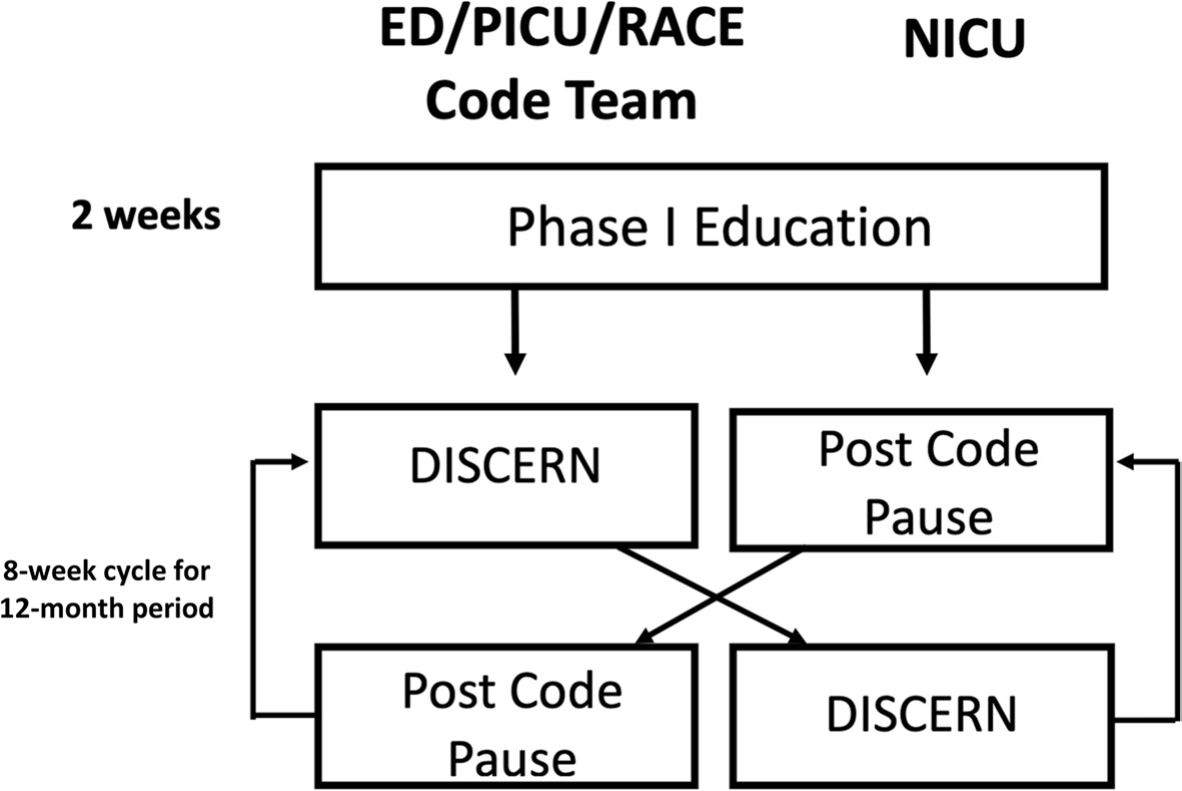
Prospective cross-over study design

The debriefing tools were studied via a prospective crossover design over a 12-month period from February 2019 to January 2020 in accordance with the Revised Standards for Quality Improvement Reporting Excellence (SQUIRE 2.0) guidelines [17]. The two tools were implemented in eight week-long blocks in each environment with each initial eight-week block starting with a one-week training period in the first two crossovers (Fig. 1). A total of six, eight week-long blocks took place over the study period for each debriefing tool. The environments using the same tool during the same time period were divided into two groups: 1) Neonatal Intensive Care Unit (NICU) and 2) Emergency Department (ED), Pediatric Intensive Care Unit (PICU), and Code Blue team, to minimize risk of cross-contamination. Members of the ED, PICU, and Code Team regularly interact in responding to events that may trigger a debriefing. The NICU interacts far less with the other teams.

### Training

The study began in two phases; Phase One entailed two weeks of education and 6 weeks of using debrief tool 1 (either DISCERN or PCP) and phase two entailed two weeks of education and 6 weeks of using the debrief tool that was not used in phase one (Fig. 1). Teams underwent a two week training period during the first two crossover blocks in the form of a PowerPoint presentation that was delivered to multiple interprofessional teams within the study locations (NICU, PICU, ED, and Code Blue team). The objectives of the training was to introduce team members to the core concepts of the post-resuscitation debriefing, its importance, as well as the plan and design of the study. During the training, each debriefing tool was reviewed as well as criteria for triggering a debrief, who can trigger a debrief, and reviewing the post-debrief surveys. Team members had a chance to review the debrief tools and ask any questions they had during training sessions. Reminders to complete debriefings took place at safety huddles at respective clinical units led by nurse educators, quality nurses or clinical managers.

### Debriefings

Debriefings were triggered in all environments for any intubation, resuscitation, event deemed to be a serious and unanticipated patient outcome, or at request by any team member for a distressing situation. Debriefs could be triggered by any of the responding healthcare professionals who felt that the situation warranted a debrief and could be led by any of the healthcare professionals participating in the critical event (e.g. physicians, registered nurses, fellows, residents, allied health providers).

Debriefings took place some point after the triggering clinical event, ranging from a few minutes following the event to a few hours after the event. Teams were able to choose a time when as many members of the clinical team as possible were available, prior to the team’s change of shift. Therefore, all debriefings aimed to be hot debriefings [11] and consisted from anywhere between five to 10 team members. The debrief leader used a copy of the debriefing tool being used, both for the purposes of facilitating the debriefing and allowing for data collection such as time to debriefing, the length of the debriefing, why the debriefing was triggered, and who attended the debriefing. The PRD tool itself was physically attached to the code cart or kept in an envelope outside of the ED resuscitation room for ease of access. The PRD tool itself was filled out by a team member who volunteered to serve as a scribe and responses were handwritten for convenience in the clinical environment. Reviews of any process or systems improvement opportunities that came up through the PRD were additionally placed on the unit’s continuous quality improvement (CQI) board and reviewed at safety huddles as part of the hospital’s CQI mandate.

### Post-debriefing surveys

Post-debriefing, team members filled out a paper or online survey consisting of Likert scale-based questions. QR codes were available so that team members could access the survey virtually via REDCap. Surveys consisted of a total of 10 questions, beginning with information on participant demographics which included number of briefings by location (ED, PICU, NICU, Code Blue team), the situation triggering the debrief, the role of the individual requesting the debrief, the roles of each of the respondents participating in the debrief by PRD tool. A seven point Likert scales were used to gather information on participant evaluation of each tool, with 1 being strongly disagree and 7 being strong agree to the following categories: tool was easy to use, tool supported the debriefing process, number of newly identified opportunities to improve, comfort in participating in the debrief process as well as the extent to which the debrief took supported emotional wellbeing, team dynamics, quality improvement and patient safety, clinical education, and other domains. Descriptive statistics were used to analyze survey responses in order to describe debrief-triggering events, participant demographics, and perceived utility of each PRD tool. T-tests were also used to compare the mean ratings of between PCP and DISCERN groups, with a pre-determined *p*-value of < 0.05 indicating a statistical difference between groups.

A thematic analysis was conducted by two independent reviewers over the course of three months. The data from the DISCERN and PCP debriefing tools were each manually coded by the two reviewers. The codes were then reviewed in collaboration between reviewers. The two separate debriefing tools, each with the questions of what went well and where improvements could be made, were independently examined. The data points from this process ultimately informed the generation themes. The debrief tools including different questions and their responses from each debriefing tool were combined together during the thematic analysis to ultimately generate the six themes. This was done to gain an understanding into the distribution of the themes across the total number of debriefing tools completed.

## Results

A total of 55 DISCERN debrief tools and 59 PCP debrief tools were completed over a period of 12 months between February 2019 and January 2020, resulting in an overall total of 114 debriefings over this time period. A total of 655 total survey responses were completed, 327 (49.9%) being in response to use of the PCP tool and 328 (50.1%) being for the DISCERN tool.

The majority of the debriefing tools (60.2%) were completed in the Emergency Department, followed by the PICU/Code Blue Team (25.0%) with the NICU completing the least number of debriefing tools (14.2%). There was participation from an interprofessional group with representation across multiple disciplines including physicians (17.1%), nursing staff (42.4%), learners (fellows, residents and & medical students) (21.1%), respiratory therapy (9.3%), child-life specialists (3.4%), social workers (2.1%), and other team members (3.7%) (Table 1).

**Table 1:** Post-resuscitation debriefing tool survey responses by location and participants

	PCP (%)	DISCERN (%)	TOTAL (%)
Total entries	*n* = 327	*n* = 328	*n* = 655
LOCATION			
ED	178 (54.4)	216 (65.8)	394 (60.2)
PICU	91 (27.8)	73 (22.3)	164 (25.0)
NICU	58 (17.8)	35 (10.7)	93 (14.2)
Wards	–	4 (1.2)	4 (0.6)
DEBRIEF REQUEST ROLE			
Staff Physician	152 (46.5)	181 (55.2)	333 (50.8)
Nurse	108 (33.0)	60 (18.3)	168 (25.6)
Resident/Fellow	19 (5.8)	27 (8.2)	46 (7.0)
RT	9 (2.8)	2 (0.6)	11 (1.7)
Pharmacy	1 (0.3)	0 (0.0)	1 (0.2)
Educator	9 (2.8)	2 (0.6)	11 (1.7)
Multiple team members	20 (6.0)	36 (11.0)	56 (8.6)
Other (e.g. child life specialist, manager, not reported)	9 (2.8)	20 (6.1)	29 (4.4)
RESPONDENT ROLE			
Staff Physician	59 (18.0)	53 (16.2)	112 (17.1)
Nurse	144 (44.0)	134 (40.9)	278 (42.4)
Resident/Fellow	57 (17.4)	72 (22.0)	129 (19.7)
RT	30 (9.2)	31 (9.5)	61 (9.3)
Pharmacy	4 (1.2)	2 (0.6)	6 (0.9)
Child Life	10 (3.1)	12 (3.7)	22 (3.4)
Social Work	6 (1.8)	8 (2.4)	14 (2.1)
Student (medical, nursing, RT)	6 (1.8)	3 (0.9)	9 (1.4)
Other (NP, medical team, unit nursing, not reported)	11 (3.5)	13 (4.0)	24 (3.7)

Physicians requested the debrief in the majority of cases (50.8%), followed by nurses who represented 25.6% of debrief requests. The remaining 14.2% of debrief requests came from residents/fellows, respiratory therapists, pharmacists, educators, and other members of the allied health team. A debrief could be triggered by any of the responding care providers. The goal was that a debrief would be triggered in any of the environments for any intubation, any resuscitation and any event deemed to be a serious or unanticipated patient outcome. Team members also requested debriefs for any distressing situations. This was most often seen in response to a patient death regardless if it was expected or not. The majority of the debriefs were triggered for a resuscitation or at the team’s request (Table 2). Given the format of the debriefing tool, it is not known what precipitated the team requesting a debrief. The average number of attendees per debrief was 5.4 individuals (5.4 for PCP and 5.3 for DISCERN). Average time from event triggering debrief was 70.4 min for PCP and 83.9 min for DISCERN (*p* = 0.2). The average duration of debriefings overall was 14.2 min. A significant difference was found in overall length of debriefings between PRD tools, with PCP debriefs lasting an average of 18.1 min while DISCERN debriefs lasted an average of 11.1 min (*p* = 0.0003). A greater variation in length of debriefing by location was observed when PCP was used with lengths of the debriefings themselves also varied by location, with code team debriefings taking an average of 13.5 min (*n* = 2), PICU debriefings taking an average of 23.6 min (*n* = 8), ED debriefings taking an average of 14.2 min (*n* = 16) and NICU debriefings lasting an average of 18.7 min (*n* = 8). However, when DISCERN was used, there was less variation in average debriefing length of time based on location, with average times of 10 min for the code team (*n* = 1), 11 min in the PICU (*n* = 10), 11.9 min in the ED (*n* = 27), and 8.8 min in the NICU (*n* = 5).

**Table 2:** Post-resuscitation debriefing tool use by reason for debriefing

	**Code Team**		**ED**		**NICU**		**PICU**		**Total**	
	**PC Pause (%)**	**DISCERN (%)**	**PC Pause (%)**	**DISCERN (%)**	**PC PAUSE (%)**	**DISCERN (%)**	**PC Pause (%)**	**DISCERN (%)**	**PC Pause (%)**	**DISCERN (%)**
**Number of debriefs**	*n* = 3	*n* = 2	*n* = 31	*n* = 34	*n* = 11	*n* = 7	*n* = 14	*n* = 12	*n* = 59	*n* = 55
**Team request**	1 (33.3)	–	9 (29.0)	10 (29.4)	4 (36.4)	–	6 (42.9)	4 (33.3)	20 (33.9)	14 (25.5)
**Resuscitation**	2 (66.7)	2 (100.0)	13 (41.9)	14 (41.2)	4 (36.4))	1 (14.3)	2 (14.3)	6 (50.0)	21 (35.6)	23 (41.8)
**Intubation**	–	–	4 (12.9)	3 (8.8)	2 (18.2)	2 (28.6)	2 (14.3))	2 (16.7)	8 (13.6)	7 (12.7)
**Unanticipated outcome**	–	–	3 (9.7)	–	1 (9.1)	1 (14.3)	2 (14.3))	–	6 (10.2)	1 (1.8)
**Other (unreported, brain death, end of life care, multiple reasons)**	–	–	2 (6.5)	7 (20.6)	–	3 (42.9)	2 (14.3)	–	4 (6.8)	10 (18.2)

When it came to participant evaluation of debrief tools, the largest differences were seen in perceived amount of emotional support offered by the PRD tool, debriefing tool support of team dynamics, as well as to what extent the tool supported clinical education. 65.2% of participants found that PCP provided emotional support while only 50% of respondents reported emotional support from DISCERN (*p* < 0.005). In terms of PRD tool support of team dynamics, 89.5% of respondents reported that PCP supported team dynamics versus 84.8% for DISCERN (*p* < 0.05). PCP was also found to strongly support clinical education (61.2% vs 56.7% for DISCERN). There were no significant differences reported in ease of use, support of the debrief process, number of identified new opportunities to improve, or comfort in making comments/raising questions during the debriefing between tools (Table 3).

**Table 3:** Participant evaluation of debrief tool

	**PC PAUSE (%)**	**DISCERN (%)**
**This survey was easy to use**		
1 – Strongly disagree	0 (0.0)	0 (0.0)
2	0 (0.0)	0 (0.0)
3	0 (0.0)	2 (0.6)
4 – Neutral	9 (2.8)	9 (2.8)
5	33 (10.1)	24 (7.4)
6	137 (42.0)	128 (39.4)
7 – Strongly agree	147 (45.1)	162 (49.8)
**The debriefing tool strongly supported the debriefing process**		
1 – Strongly disagree	0 (0.0)	0 (0.0)
2	0 (0.0)	0 (0.0)
3	2 (0.6)	2 (0.6)
4 – Neutral	13 (4.0)	13 (4.0)
5	37 (11.4)	28 (8.6)
6	140 (42.9)	129 (39.4)
7 – Strongly agree	134 (41.1)	155 (47.4)
**Comfort making comments/raising questions during debriefing**		
1 – Strongly disagree	1 (0.3)	0 (0.0)
2	0 (0.0)	2 (0.6)
3	3 (0.9)	1 (0.3)
4 – Neutral	12 (3.7)	16 (5.0)
5	35 (10.8)	22 (6.8)
6	79 (24.4)	98 (30.3)
7 – Strongly agree	194 (59.9)	184 (57.0)
**Number of identified new opportunities to improve**		
None	62 (18.9)	36 (11.7)
1	79 (24.1)	84 (27.2)
2	105 (32.0)	94 (30.4)
3 or more	82 (25.0)	95 (30.7)
**Debriefing tool supported emotion support**		
0 – No	113 (34.8)	164 (50.0)
1 – Yes	212 (65.2)	164 (50.0)
**Debriefing tool supported team dynamics**		
0 – No	34 (10.5)	50 (15.2)
1 – Yes	291 (89.5)	278 (84.8)
**Debriefing tool supported quality improvement/patient safety**		
0 – No	36 (11.1)	43 (13.1)
1 – Yes	289 (88.9)	285 (86.9)
**Debriefing tool supported clinical education**		
0 – No	126 (38.8)	142 (43.3)
1 – Yes	199 (61.2)	186 (56.7)
**Debriefing tool supported other**		
0 – No	320 (98.5)	313 (95.4)
1 – Yes	5 (1.5)	15 (4.6)

### Thematic analysis

Thematic analysis of qualitative responses to free text were also analyzed to better understand differences between tools and how this contributes to PRD tool utility. There were comments that were not able to be coded as they directly pertained to the critical event such as documenting what type of event, or very specific courses of action taken during the event, which have not been reported for confidentiality purposes. Additionally, there were comments that were difficult to interpret due to lack of context or the writing was not able to be deciphered, which were also excluded from the thematic analysis.

Thematic analysis of the survey results for each PRD tool revealed six key themes: (1) communication, (2) quality of care, (3) team function & dynamics, (4) resource allocation, (5) preparation and response, and (6) support.

Communication and team functioning were commonly identified in both the areas of strength and areas of improvement. There was little consistency among the survey responses in terms of themes of what went well and what could be improved. Themes were identified as being a strength in one critical event and then found to a weakness in a subsequent critical event. Commonly, closed loop communication, team leader identification and identification of the critical event were highlighted as areas of both strength and weakness depending on the scenario. A few examples of the data obtained from the debriefing tools broken down between strengths and areas of improvement are include Table 4.

**Table 4:** Qualitative responses by PRD tool by theme

Theme	PCP	DISCERN
Items identified as done well		
Communication	“excellent closed loop communication” “direct [and] focus[ed]” “summarize[d] plan” “procedure pause prior to intubation”	“clear communication about meds needed” “people talking loud enough for recorder to hear” “closed loop communication” “calm in the room”
Team Function	“shared mental model of priorities” “clear instructions, loud succinct team leader” “utilized interdisciplinary team”	“organize resuscitation, all roles were assigned” “clear leader, documenter knew what was happening” “quick team assembled”
Quality of Care	“respectful of cultural practice” “able to get family in”	“updating parents throughout” “advocated for patient to have dad present” “mom’s request being honoured by the ED team”
Equipment	“quick provision of meds [and] equipment”, “adequate equipment”	“use of language line…code blue button use” “use of FAST” “CT quick”
Preparation	“early identification of a sick patient” “good anticipation” “quick, effective recognition of [a] critical situation”	“initiated quickly, appropriate assessment of intervention” “everyone aware prior to patient arrival…plan in place prior…” “very rapid response by the code team” “prompt treatment”
Support	“support each other” “respected student[s]” “juniors well supported”	“bedside teaching awesome” “respected the space for learners” “junior learner: well supported, good educational value”
Identified areas of improvement		
Communication	“communication between attendings not clean” “better closed loop communication” “better system available to communicate for OR and ISR” “SBAR to PICU” “too noisy at the nursing station, nurses & MDs talking outside room” “speak up if something not as it should be (front line person)”	“More vocalization” “[remember] close loop and clear who lead is” “recap presentation for others” “more closed loop communication in terms of plan & future interventions”
Team Function	“better documentation of orders” “[involvement] of security to help manage crowd” “PACE very disorganized” “more clear role description of resus leader” “clear team leader”	“EMS blocking head of bed” “the leader role was not clear” “clear role definition” “way to identify ER staff/position for PICU coming in to assist”
Quality of Care	**“**cultural awareness…access to ethics” “assign 1 person to communicate with parents” “ongoing updating to the family during resuscitation…who’s role?”	“child life should have been allowed in” “parents could’ve been brought in sooner” “mom should have been filled in prior [to] coming into room” “support for mom”
Equipment	“curved blade would have been helpful” “computer to chart would have been handy” “no 3% saline nebs in the trauma rooms”	“no emergency UVC kit on code card” “improvement with implementing epileptic order set” “trouble getting team paged” “code pink not announced officially by button”
Preparation	“delay in consult service arrival” “plan to give alert for exchange [transfusion] to make sure everything is ready so when we need blood, will be ready” “call code whenever team wants” “BP/temp sooner” “move to resus sooner”	“ > 2 h prep time [for exchange transfusion]…next time to initiate earlier” “prepare all emergency medications…anticipating for the worst” “delayed identification of clinical status”
Support	“Not to be afraid, hesitant to activate [code]” “continue to provide support to rotating learners”	“positioning of learners” “recognized learners in situation”

It was also subjectively noted that it was easier to identify more consistent themes from the DISCERN tool as the questions were more open-ended, whereas the PCP tool seemingly allowed for broader interpretation of the event and therefore had more varied, albeit shorter, responses. Overall, it was easier to identify specific themes using the DISCERN tool, while the PCP tool’s multiple focused questions allowed for easier organization and classification of themes.

For example, communication was featured as a dominating theme throughout the debriefing surveys. The response to the questions asked in the DISCERN tool were less descriptive – simply “closed-loop communication” or “good communication”. For the same theme of communication, the PCP tool included response such as “clear instructions, succinct team leader” in one of the survey responses or “better introduction of people present” in another response to the same questions of what went well and what could be improved. Although communication was featured as a theme throughout the survey responses, it was both identified as a strength and as an area of improvement of different critical events. For example, from the PCP responses, one event identified “excellent closed loop communication […] calm approach” as an area where things went well, while another reported “improve communication…[use] closed loop communication” as an area of improvement. This was also evidenced in the DISCERN study responses.

A very common theme identified in both the DISCERN and PCP surveys was the functioning of the responders as a team during the critical event. Team dynamics was both in reference to communication throughout the event, as well as leadership and role clarity. Again, in both tools this theme was identified as a strength in some events and as an area for improvement in others. In one of the events that was debriefed using the DISCERN tool, it was noted “roles were known and established early […] everyone [was] calm and acted quickly with clear communication”. But it was noted in debriefs using both the DISCERN and PCP tools that areas of improvement included “clear role definition…[trauma team lead] did not identify self” and “better introduction of people present” from each tool, respectively. The role clarity amongst responders and decision making were strong points as well as weak points depending on the event and the location.

## Discussion

The process of debriefing has been shown to play an important role in identifying and addressing human factors in the healthcare setting as they pertain to patient safety [15]. It has also been shown to be recognized as an important aspect of patient care, quality improvement, and medical education by healthcare providers, but is difficult to implement into the clinical setting due to poor standardization, beliefs surrounding a certain degree of training needed to facilitate appropriate debriefings, as well as workload demands and time constraints. Furthermore, the highly varied environments in which PRD has been studied, ranging from medical education and simulation, to evaluation and assessment, to clinical environments which differ in workflow and logistical constraints further compound the difficulty of implementing a post-resuscitation debriefing culture into pediatric acute care environments, despite published need and motivation to do so [2]. The use of PRD tools have been cited as potential solutions to address the barriers towards implementing PRD practices, however, lack of direct comparison between PRD tools currently makes it impossible for institutions to adopt an evidence-based PRD tool that meets the needs of their team and clinical environment. The literature recommends focusing on core principles including clarify the objectives for debriefing as this has the potential to help in identifying both the method(s) and outcomes measures of importance when selecting debriefing tools and methods [11].

As a result, the objective of the study was to directly compare two PRD tools, DISCERN and PCP, in an effort to determine which tool is the most effective to facilitate post-critical event debriefs in acute care settings within a tertiary care children’s hospital. The two debriefing tools, DISCERN and PCP, were used in four different settings (NICU, PICU, ED and pediatrics Code Blue team) with multidisciplinary teams including but not limited to physicians, nursing staff, pharmacists, and medical learners of various stages, with the goal of facilitating a debriefing discussion following a critical event. The results of this study provide novel and fascinating insight into the role of debriefing in a clinical setting, the types of events and environments in which debriefs are requested by members of an interdisciplinary healthcare team, as well as the components of a PRD tool that contribute towards meaningful and efficient debriefings.

Given that a formal PRD process did not exist at our institution prior to this study, the process of PRD had to be implemented in order to carry out this study. The process of this implementation was assessed with regards to timing of training employees, resources, time of debriefings themselves, and other logistical considerations for implementation of a PRD tool. It was previously been described that the majority of healthcare workers believe that some formal training is required for the process of PRD and that time of debriefings are a barrier to PRD [2]. Training was easily carried out through the hospital wide continuous quality improvement initiative by Nurse Educators and Quality Nurses of the various clinical units and also with reminders at daily safety huddles at clinical units. Making PRD tools easily accessible to team members was also found to be feasible by having physical copies attached to code carts and in an envelope outside the resuscitation room in the ED. With regards to length of the debriefings themselves, the average duration of debriefings using PCP was found to be 18.1 min, while DISCERN debriefings lasted an average of 11.1 min, which constituted a significant difference between PRD tools, so this may be factor to consider when choosing a debrief tool. Overall, the average time of all debriefings was quite short, lasting an average of 14.2 min, and reinforces that PRD is feasible and realistic for acute clinical areas.

Although there were a wide variety of distressing situations which triggered debriefs captured by this study, including resuscitations and unanticipated outcomes resulting in the death of patients, the majority of debriefs were triggered in response to a patient death regardless of whether it was expected or not. Both tools were found to be useful in providing emotional support as well as identifying areas for improvement on individual, team, and systems levels in these scenarios. This speaks to the need for teams to have access to some kind of format for debrief in healthcare settings for emotional wellbeing of team members as well as to support quality improvement and patient care. Team members are astute when it comes to recognizing when debriefing is required. Creating ease of access to debrief resources, formats, and/or PRD tools can help facilitate a culture where debriefs are incorporated into daily practice.

Despite the differences that exist between the content and structure of DISCERN and PCP, the only significant differences in user-rated experience were found to be in the level of emotional support that the tool seemed to help foster as well as the support of clinical education.

Notably, the majority of debriefs, using either tool, were triggered in the Emergency Department in response to a critical event deemed to require debriefing by a team member involved in the case. The ED environment poses particular challenges with regards to practical considerations during debriefs including significant time restraints, the potential for a wide variety of triggering events including novel scenarios never previously encountered by a team, as well as a team that is likely to change more frequently than other departments, thus posing additional challenges when it comes to debrief comfort and emotional support required. Despite demanding, high acuity, and fast-paced environments, both tools were found to be equally easy to use, as well as found to equally support the debrief process, identify new opportunities for improvement, and contribute to participant comfort when debriefing. This lends further support to the idea that regardless of the tool, some kind of debrief framework is helpful to teams in both encouraging debriefing and in helping to facilitate the process and yielding valuable results.

The DISCERN tool, while helpful with identifying what went well and what could be improved, actually did not address the team members’ overall wellbeing following the event. In reviewing the comments from the DISCERN tool, there were no responses commenting on how the staff were doing post-event. Team support was identified multiple times as a strength, but without any further details describing what exactly those elements of team support were.

The PCP specifically asked how the responders were doing after the event and what would be needed the healthcare professionals to safely return to work. These questions highlighted emotions such as “tired”, “proud”, “hard to go back to work when it’s non-acute things after [resuscitation]” and “emotionally okay”. It also helped to elicit actionable items for post-resuscitation care including “more reasonable clinical load when carrying the front-line pager” or “check-in with each other…”. It not only provided a safe environment for team members to debrief on their emotions following a high-stakes event, but also an opportunity to discuss how staff support during and after these events can be improved.

Thematic analysis revealed that while the DISCERN tool’s broad questions (ie. What went well? What could be improved?) allowed healthcare professionals to reflect on the event, it did not always fully explore some of the themes raised. It did, however, give the responders a space to speak about anything that arose that they felt the need to discuss post-event. PCP provided a more organized approach to the debrief and was able to direct the conversation to key areas of debriefing. There was the opportunity to discuss what went well during the critical event, but the areas of improvement were further broken down. Specific questions allowed for comment on the availability of interventions, medications and equipment in addition to a question about general improvement. Although the answers to the specific prompts were briefer, they were more focused and allowed for the pulling of actionable items for improvement in the response of critical events. PCP importantly also contains a section on the mental and emotional wellbeing of healthcare professionals post-event. This is something that may get missed in a debrief as healthcare professionals focus on what went well and what could be improved. This was evidenced in the DISCERN tool as there were no comments pertaining to how the healthcare professionals were doing after the event or what could be added to support them post-critical events.

With regards to the thematic analysis, a very common theme identified in both the DISCERN and PCP surveys was the functioning of the responders as a team during the critical event. Team dynamics was both in reference to communication throughout the event, as well as leadership and role clarity. Again, in both tools this theme was identified as a strength in some events and as an area for improvement in others. Although themes were both identified as a strength in one scenario, and then as an area of improvement in the next, it is important to note that the scenarios that were being debriefed were not standardize and neither were the responding healthcare professions. This resulted in different strengths and areas of improvement being described in each of the debrief tools. It was difficult to identify consistent themes of strengths or weakness due to the variation of location and type of critical event in addition to different healthcare providers responding to said event.

Additional themes, including equipment availability and preparation were emphasized as being very important to a resuscitation across the debriefing tools. This was again brought up as both a strength and as a weakness dependent on the scenario being debriefed. Although, the DISCERN tool also provided specific actionable items for equipment malfunctions and proposed equipment changes, it is sometimes lost in the other comments or not fully explored. It may be due to the debrief documenter only writing down key works “glidescope stylet” or “delay of urgent x-ray”, but never explored any further. Again, the broad nature of the question “what could be improved?” as used in the DISCERN tool provides a blank slate for possible responses. It was noted that often it actually resulted in vague and non-descriptive answers.

This is contrasted with the PCP tool, which used very specific questions as part of the debriefing process. It specifically asked both what interventions the team wished they had offered as well as whether the team was satisfied with the availability of the medication and equipment. This resulted in responses such as “room was set up appropriately… [but] computer to chart would be helpful” or “curved [laryngoscope] blade would have been helpful”. This is helpful in providing actionable items for improvement on the overall resuscitation process. If the debrief tool is to be used for identification of areas of improvement, providing specific questions in order to both lead the discussion and provide an organized documentation may prove to be useful.

The primary objective of this study was to assess user preference with regards to utility of two PRD tools, DISCERN and PCP, based on personal, situational, environmental, and team-based factors, with the ultimate goal of this initiative being to institute a children’s hospital-wide debriefing tool for PRD. Secondary aims included determining whether there were differences in the quality, subject matter, and types of feedback garnered from these different tools and potential implications on quality improvement and patient safety. Given the dynamic nature of a clinical environment, there were a variety of factors that could not be controlled in this study, including how long after the critical event the debriefing itself took place, which team members were present at the debrief, the space in which the debrief took place, and the amount of time that was available for the debrief to take place, which limits the standardization of the debriefing process. Furthermore, given the nature of events being discussed, there was content in the debriefs that was not included in analysis to maintain patient confidentiality. Finally, although one of the secondary aims of the study was to assess the implications of the debriefing on quality improvement and patient safety, which was done by comparing responses between PRD tools, whether or not identified areas of improvement were translated into practice and the ease of implementing these changes was not assessed through this study.

In medicine, the wellbeing of the medical team or responding team is not often thought about as being critical to the response of a resuscitation. The goal of a debriefing tool should be two-fold: both to identify the strengths and areas of improvement of the event, but also to allow the responding staff to express how they are feeling post-event. As a result, it is recommended that some form of debrief tool be made available and accessible to healthcare providers to help facilitate these discussions. The implementation of a structured PRD tool can remove many of the barriers to the process of debriefing and thus help facilitate discussion, improve emotional support and comfort for participants, and lead to more consistent debriefs across teams and settings. This study found that a PRD tool is feasible to implement with respect to both formal training of healthcare workers on the use of each tool and the purpose and importance of PRD as well as the time of debriefings themselves, both of which have been previously identified as barriers towards implementing PRD [2]. Based on the results of this study, it is recommended that an institution or setting specific PRD tool be chosen based on institution and team values and preferences and demands including team dynamics, constraints of the environment or setting such as time restrictions, and especially the degree of emotional support deemed to be valuable from a quality improvement and patient safety perspective. Other options including having a variety of PRD tools available with the option for teams to choose a debrief tool to use based on the triggering situation such as tools available for times when more emotional support is deemed necessary such as after an unexpected death versus times when more quality improvement/patient safety ideas are required such as after an unexpected outcome.

## Data Availability

All data generated or analyzed during this study are included in this published article.

## Abbreviations

PRD: Post-resuscitation debriefing
PCP: Post-code Pause
NICU: Neonatal Intensive Care Unit
PICU: Pediatric Intensive Care Unit
ED: Emergency Department
SQUIRE 2.0: Revised Standards for Quality Improvement Reporting Excellence
